# AML1/ETO accelerates cell migration and impairs cell-to-cell adhesion and homing of hematopoietic stem/progenitor cells

**DOI:** 10.1038/srep34957

**Published:** 2016-10-07

**Authors:** Marco Saia, Alberto Termanini, Nicoletta Rizzi, Massimiliano Mazza, Elisa Barbieri, Debora Valli, Paolo Ciana, Alicja M. Gruszka, Myriam Alcalay

**Affiliations:** 1 Department of Experimental Oncology, Istituto Europeo di Oncologia, Via Adamello 16, 20139, Milan, Italy; 2 Department of Pharmacological and Biomolecular Sciences (DiSFeB), University of Milan, Via Balzaretti 9, 20133, Milan, Italy; 3 Department of Oncology and Hemato-Oncology, University of Milan, Via Festa del Perdono 7, 20122, Milan, Italy

## Abstract

The AML1/ETO fusion protein found in acute myeloid leukemias functions as a transcriptional regulator by recruiting co-repressor complexes to its DNA binding site. In order to extend the understanding of its role in preleukemia, we expressed AML1/ETO in a murine immortalized pluripotent hematopoietic stem/progenitor cell line, EML C1, and found that genes involved in functions such as cell-to-cell adhesion and cell motility were among the most significantly regulated as determined by RNA sequencing. In functional assays, AML1/ETO-expressing cells showed a decrease in adhesion to stromal cells, an increase of cell migration rate *in vitro*, and displayed an impairment in homing and engraftment *in vivo* upon transplantation into recipient mice. Our results suggest that AML1/ETO expression determines a more mobile and less adherent phenotype in preleukemic cells, therefore altering the interaction with the hematopoietic niche, potentially leading to the migration across the bone marrow barrier and to disease progression.

Approximately 12–15% of cases with adult acute myeloid leukemia (AML) carry the (8;21) translocation, which fuses the *AML1* (also known as *RUNX1*) and *ETO* (otherwise *RUNX1T1* or *MTG8*) genes and results in expression of the AML1/ETO chimeric protein^1^. The published expression data demonstrate that AML1/ETO expression induces a distinct gene expression profile that involves the regulation of haematopoietic transcription and anti-apoptotic factors, DNA repair genes and tumor suppressors^2^,^3^. Hence, the expression of AML1/ETO blocks differentiation *in vitro* of several hematopoietic lineages^4^, increases self-renewal of hematopoietic progenitors^5^ and induces a myeloproliferative disorder in mouse models^6^, but it is insufficient for the induction of leukemia *in vivo*^7^, indicating the necessity of additional mutations for the disease onset.

The identification of functions that are disrupted by fusion proteins during the preleukemic phase is crucial for understanding the molecular basis of leukemia progression. Both normal hematopoietic stem cells (HSC) and (pre)-/leukemic stem cells (LSC) rely on tightly regulated interactions with the microenvironment or “niche” for their survival^8^. Several studies have reported that specific molecules, including CD44, osteopontin and E-selectin, are relevant for LSC-niche interactions. Interestingly, genes encoding for proteins involved in cellular adhesion and/or migration were found to be common targets for the AML1, AML2 and AML3 transcription factors^9^, and aberrant expression of adhesion molecules has been described in acute lymphoblastic leukemia cases carrying the TEL/AML1 (or ETV6/RUNX1) fusion gene^10^. Moreover, expression of CD44^11^, VLA-4^12^ and LFA-1(or CD11a)^13^ adhesion molecules has been found to be directly regulated by AML1/ETO.

In order to study AML1/ETO’s role in preleukemia, we exploited a murine immortalized pluripotent hematopoietic stem/progenitor (HSPC) cell line, EML C1 (referred to as EML from here on), which can be induced to differentiate into both the lymphoid and myeloid lineages upon appropriate cytokine treatment^14^. Two AML1/ETO-expressing EML clones were generated by retroviral transduction and characterized for their growth and differentiation properties. The analysis of gene expression by RNA sequencing showed that cell migration and cell-to-cell adhesion were among the most important functions regulated by the fusion protein, suggesting that interaction with the microenvironment may be altered in preleukemic precursors expressing the fusion protein. This manuscript explores the effects of AML1/ETO expression on migration and adhesion properties in hematopoietic cells *in vitro* and homing/engraftment *in vivo*.

## Results

### AML1/ETO regulates the expression of genes involved in cell migration and adhesion

The murine HSPC cell line EML^15^ was used to investigate AML1/ETO function in this study. EML cells correspond to short-term HSC as demonstrated by FACS analysis of stem-cell surface antigens (Supplementary Fig. S1), and have the capacity to undergo multi-lineage differentiation *in vitro* when treated with appropriate cytokines^16^. In particular, as previously described^15^, myeloid differentiation (attested to by an increased expression of Mac-1 and Gr-1 myeloid markers, and a decreased level of Sca-1 and cKit stem cell markers) can be achieved by treatment with all-trans retinoic acid (atRA) and IL-3 for 3 days, and subsequently with GM-CSF for 5–8 days, and monitored by flow cytometry^16^.

**Figure 1:**
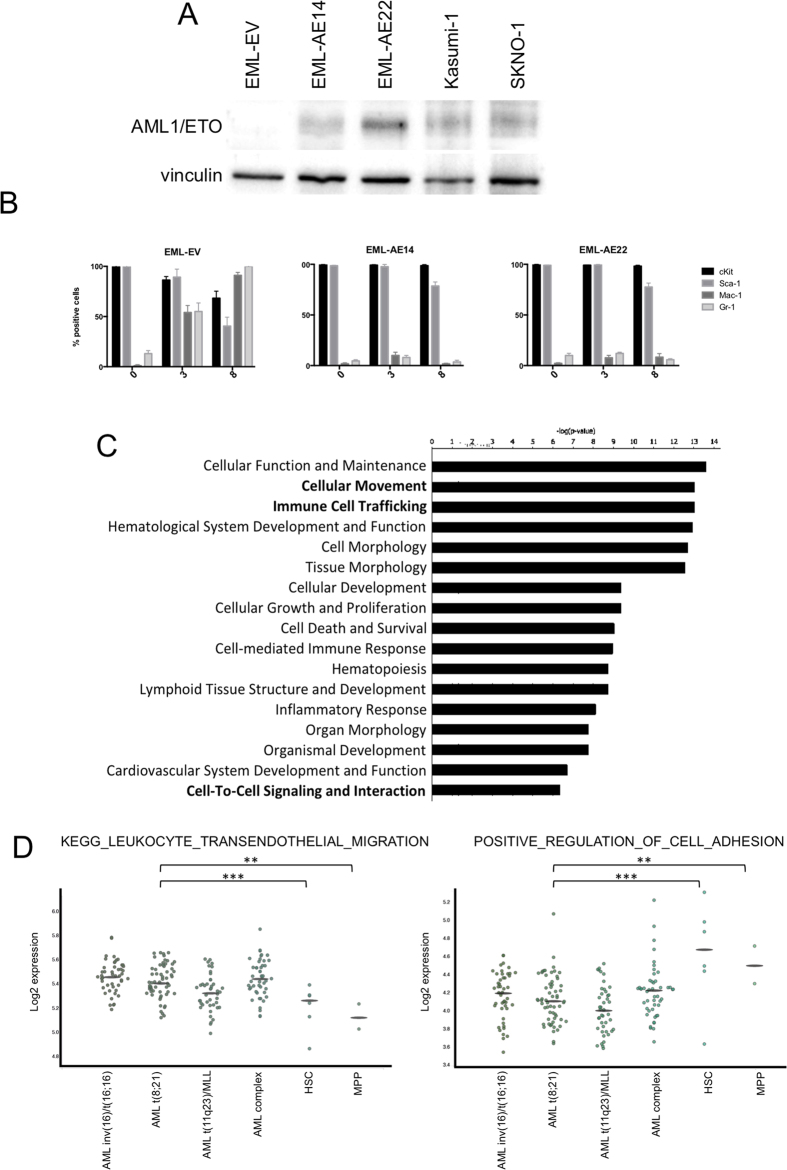
AML1/ETO regulates genes involved in cellular migration and adhesion. (**A**) AML1/ETO protein levels in EML-AE clones used in this study were compared to those in patient-derived cell lines Kasumi-1 and SKNO-1 by Western blotting with an anti-ETO antibody. Sample loading was controlled by detection of Vinculin. (**B**) Kinetics of myeloid differentiation as measured by FACS analysis of cKit, Sca-1, Mac-1 and Gr-1 surface in untreated (0 days), atRA (3^rd^ day of treatment) and GM-CSF (8^th^ day of treatment) treated EML-EV, EML-AE14 and EML-AE22 cells. (**C**) Ingenuity Pathway Analysis (IPA) classification of functions enriched in the list of genes regulated in EML-AE22 cells compared to EML-EV cells identified by RNA-seq. (**D**) BloodSpot plots showing the expression data of public adhesion and migration signatures in AML subtypes and normal HSC/MPP cells. For each signature, the mean expression values for all samples in all datasets were computed and reported as dots in y-axis. Averaged values represented the expression of a signature for each sample. Statistical analysis was performed on the distribution of these values between the AML t(8; 21) dataset and the normal HSC dataset.

The full-length AML1/ETO fusion transcript was expressed in EML cells by retroviral transduction using the PINCO-GFP vector and two clones that displayed high AML1/ETO expression (EML-AE14 and EML-AE22) were selected by serial dilution. A control cell line transduced with empty vector (EML-EV) was also generated. Western blot analysis showed that EML-AE14 and EML-AE22 cells expressed AML1/ETO protein at levels similar to Kasumi-1 and SKNO-1 - two AML patient-derived cell lines that carry the t(8;21) translocation (Fig. 1A). AML1/ETO-expressing cells showed growth characteristics similar to EML cells and did not display any cell cycle alterations, no increase in apoptosis or induction of senescence (Supplementary Fig. S2).

Studies showed that AML1/ETO-expressing cells are defective in myeloid differentiation^17^. To validate our model system, cells were treated with cytokines as described above. After 8 days of treatment whilst EML-EV cells differentiated (left panel of Fig. 1B) AML1/ETO-expressing clones showed a complete block of differentiation, as testified by the persistent expression of stem cell markers by the majority of cells with little induction of myeloid marker expression during cytokine treatment (middle and right panels of Fig. 1B). Cells kept in medium without cytokines were analyzed as well, and showed no modification of surface marker phenotype within the observation time (data not shown). The results revealed no difference between the two clones, and thus clone EML-AE22 was used throughout for further experiments, while EML-AE14 was used in selected confirmatory tests.

To further characterize the EML-AE cell lines, global gene expression was analyzed by RNA sequencing (RNA-seq). Total RNA was extracted from EML-AE22 cell and EML-EV control cells, RNA-seq libraries were generated and sequenced. 1572 genes were found to be differentially expressed in EML-AE22 cells compared to EML-EV (921 upregulated and 651 downregulated, Supplementary Table S1). RNA-seq results were validated by RT-qPCR analysis of 15 genes (Supplementary Fig. S3).

Functional classification of AML1/ETO regulated genes was performed using different analysis software, including Ingenuity Pathway Analysis (IPA), DAVID and Gene Set Enrichment Analysis (GSEA). IPA identified motility, immune cell trafficking and cell-to-cell signaling and interaction among the most enriched cellular and molecular functions (Fig. 1C), yielding a list of 194 genes (Supplementary Table S2) that included integrins, interleukins, chemokines and their receptors, adhesion molecules, actin cytoskeleton-regulatory proteins, intracellular regulatory kinases, and motility related regulators (Supplementary Fig. S4A). Coherently, the DAVID pathway analysis tool showed significant enrichment of chemokine signaling pathways, regulation of actin cytoskeleton and cell adhesion molecules (Supplementary Fig. S4B). We then used the IPA tool to analyze the gene expression profile of the AML1/ETO-expressing U937 cell line previously generated in our laboratory^3^ and found an enrichment of the same functions (Supplementary Fig. S4C).

To investigate if these functions are altered also in transcriptional profiles derived from AML patients that carry the t(8; 21), BloodSpot^18^ was interrogated. BloodSpot analyses the public expression data in all available datasets for AML subtypes and normal HSC/MPP cells and computes the mean expression values. The analysis revealed that a migration-related gene signature (“KEGG_LEUKOCYTE_TRANSENDOTHELIAL_MIGRATION”) is indeed overexpressed in all AML types analyzed, including cases with t(8; 21), as compared to both HSC and multipotent progenitors (MPP), while adhesion signature (“POSITIVE_REGULATION_OF_CELL_ADHESION”) is downregulated in the same AML subgroups (Fig. 1D).

Taken together, these results indicate that AML1/ETO regulates the expression of genes involved in migration and cell-to-cell adhesion in HSPC, and that these functions are altered in different AML subtypes suggesting they may be of relevance for disease progression.

### AML1/ETO expression impacts on motility and adhesion properties of hematopoietic *and leukemic cells in vitro*

**Figure 2:**
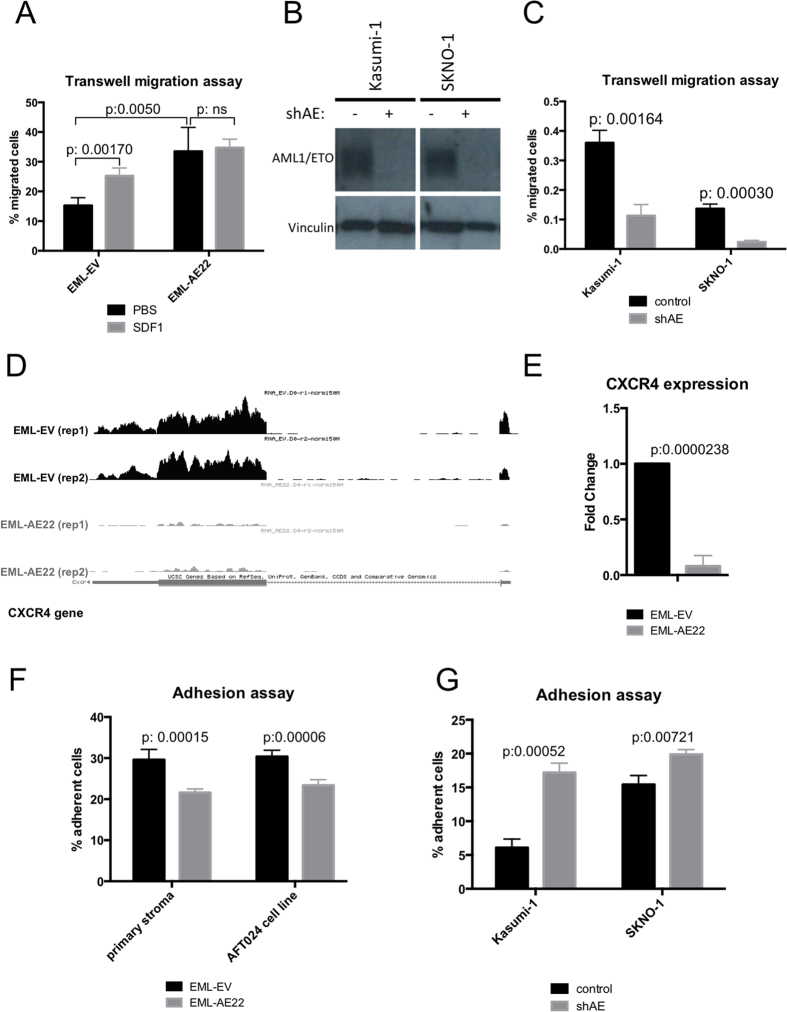
AML1/ETO expression regulates motility and adhesion of hematopoietic and leukemic cells. (**A**) Transwell migration assay of EML-EV control and EML-AE22 cell with (grey bars) or without (black bars) the addition of SDF1 to the lower chamber. (**B**) Western Blot analysis of AML1/ETO protein expression upon shRNA-mediated interference in Kasumi-1 and SKNO-1 cell lines. Vinculin was used as loading control. (**C**) Transwell migration assay on Kasumi-1 and SKNO-1 cells upon AML1/ETO interference (shAE). (**D**) RNA-seq track of EML-EV control cells and EML-AE22 cells at the *Cxcr4* gene locus. The two biological replicates used for sequencing are shown (rep1 and rep2). (**E**) *Cxcr4* expression levels were measured by RT-qPCR in EML-EV and EML-AE22 cells. Levels are relative to *Tbp* expression and normalized for expression in the control cells. (**F**) Adhesion assay of EML-EV and EML-AE22 cells on primary murine bone marrow stroma and ATF024 cell lines. (**G**) Adhesion properties of Kasumi-1 and SKNO-1 human AML cell line after AML1/ETO interference (shAE) on ATF024 cells.

A series of *in vitro* experiments was performed to investigate the migration and adhesion properties of AML1/ETO-expressing cells. EML-AE22 cells were used in transwell migration assay, in which a serum gradient was employed as an attractant for cells placed in the upper chamber. After 8 hours incubation, EML-AE22 cells showed an increased migration ratio compared to EML-EV control cells (*p*value = 0.005, Fig. 2A) and the same outcome was also seen in EML-AE14 (Fig. S5A). Conversely, shRNA-mediated depletion of AML1/ETO (Fig. 2B) in Kasumi-1 and SKNO-1 caused a decrease in transwell migration, confirming the results obtained with EML cells (*p*value = 0.00164 and 0.0003 respectively, Fig. 2C).

Stromal Derived Factor 1 (SDF1 or CXCL12) is the most important cytokine involved in homing of HSCs^19^,^20^. RNA-seq and quantitative PCR revealed downregulation of *CXCR4*, the gene encoding for the SDF1 receptor in EML-AE22 cells (Fig. 2D,E). We therefore assessed the migration rate of EML-EV and EML-AE22 cells upon addition of SDF1 to the medium in the lower chamber of the transwell. SDF1 (100 ng/ml) increased the migration of EML-EV cells by 1.6 fold compared to PBS control (*p*value = 0.017), but did not alter the migration rate of EML-AE22 cells (*p*value = ns, Fig. 2A), likely due to the encountered downregulation of CXCR4 receptor in EML-AE22 cells. This result was confirmed by repeating the assay using EML-AE14 cells (Supplementary Fig. S5A).

Several adhesion molecules (integrins, CD34, SELL, CD93, ICAM1) were found to be regulated by AML1/ETO, suggesting that the cell-to-cell adhesion could be impaired in EML-AE22 cells. EML-AE22 and EML-EV cells were plated onto a primary murine bone marrow stroma layer or onto the AFT024 fetal liver stromal cell line in order to study their adhesion capacity. After a two-hour incubation, a lower fraction of EML-AE22 cells adhered to the substrate compared to EML-EV cells, independently of the cell type used as substrate (*p*value = 0.00015 and 0.00006 respectively, Fig. 2F). This result was confirmed by repeating the assay using EML-AE14 cells (Supplementary Fig. S5B). Although to a lesser extent, the shRNA-mediated knock-down of AML1/ETO in the Kasumi-1 and SKNO-1 cell line increased adhesion to ATF024 cells (*p*value = 0.00052 and 0.00721 respectively Fig. 2G).

Taken together, these results show that AML1/ETO-expressing HSPCs display increased cell motility and a concomitant reduction in cell-to-stroma adhesion *in vitro*.

### Expression of AML1/ETO impairs homing and delays engraftment

Homing is a process by which transplanted stem cells respond to the chemoattractants secreted by different bone marrow cell types, react with the vessels within the bone marrow and migrate into the extravascular space^21^. Indeed, homing involves both migration and adhesion^20^. We speculated that altered migration and adhesion properties of the fusion-expressing cells might be translated into changes in the homing capacity of AML1/ETO-expressing HSPCs to mouse bone marrow and other hematopoietic target organs compared to control cells.

**Figure 3:**
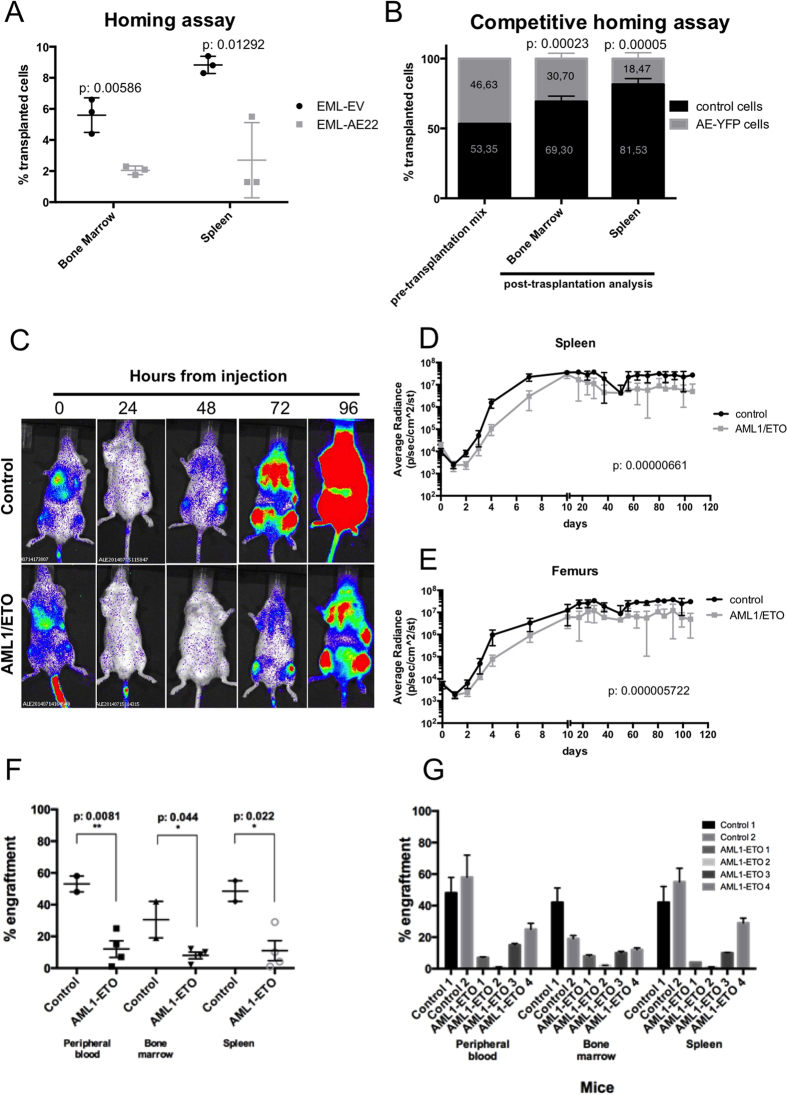
Expression of AML1/ETO impairs homing and engraftment. (**A**) Homing assay of EML-EV and EML-AE22 cells into irradiated recipient mice (three per cohort). Bars represent the percentage of eFluor^®^670–positive cells in the tissue analyzed. (**B**) Competitive homing assay of AML1/ETO-eYFP-Cre-ER Ly5.2 (AE-YFP) knock-in bone marrow cells and Cre-ER Ly5.2 control cells (three mice per cohort). The percentage of the two cellular populations in the transplanted sample is also shown (pre-transplantation mix). Bone marrow and spleen bars represent the FACS analysis of Ly5.2 cells present in each tissue. (**C**) Live bioimaging analysis of homing and engraftment of Lin-LUC-EV (control, up) or Lin-LUC-AE (AML1/ETO, down) cells transplanted into B6 Albino recipient mice (5 mice per cohort). One representative mouse per cohort is shown. Images were acquired every 24 hours after injection. (**D**–**E**) Kinetics of the engraftment of Lin-LUC2 control and AML1/ETO cells into recipient irradiated mice measured by live bioimaging. Average radiance of spleens (**D**) and femurs (**E**) for each cohort is shown. (**F**) Engraftment levels in peripheral blood, bone marrow and spleen at the end of the bioimaging experiment shown for the control and AML1/ETO-expressing cohorts. (**G**) Engraftment level in peripheral blood, bone marrow and spleen shown for individual mice.

Homing experiments were performed in several model systems. Initially, EML-AE22 or EML-EV cells stained with eFluor^®^670 injected into sub-lethally irradiated NOD/SCID mice. Sixteen-hours later, bone marrow and spleens were taken from the sacrificed animals and eFluor^®^670-positive cells were quantified by flow cytometry. A significantly lower percentage of AML1/ETO-expressing cells compared to control cells were present in the hematopoietic organs of the recipients (*p*value = 0.00586 for bone marrow and 0.01292 for spleen respectively Fig. 3A).

Competitive homing experiments allow the comparison of homing efficiencies of two cell types within the same recipient, thus decreasing technical variability. This type of assay was performed using bone marrow mononuclear cells purified from an inducible AML1/ETO-eYFP-Cre-ER Ly5.2 knock-in mouse model. These mice express both AML1/ETO and eYFP in the hematopoietic system upon nuclear translocation of Cre-ER induced by tamoxifen. Cre-ER Ly5.2 mice were used as a source of control cells. Both ablative and non-ablative experiments were performed; in the latter case, not irradiated animals with steady-state hematopoiesis were used as recipients in order to investigate homing of transplanted Ly5.2-cells into the physiological and unaltered bone marrow niche. Following dietary tamoxifen-mediated induction of Cre nuclear translocation, AML1/ETO-eYFP and control Cre-ER cells were mixed in equal quantity (pre-transplantation mix in Fig. 3B), and injected into not irradiated Ly5.1 mice. After 16 hours, recipient animals were sacrificed and FACS analysis of cells from target organs was performed. A significantly lower number of Ly5.2-positive AML1/ETO-eYFP cells homed into the bone marrow (2.26 fold less, *p*value = 0.00023) and spleen (4.41 fold less, *p*value = 0.00005) compared to Cre-ER Ly5.2 control cells (Fig. 3B), confirming the homing defect observed using EML-AE22 cells. The result was confirmed in the ablative setting (Supplementary Fig. S6A), even though a smaller difference was observed between AML1/ETO-eYFP and control cells that homed to bone marrow (1.59 fold less) and spleen (1.57 fold less). As expected, a significantly higher proportion of injected cells reached the target organs when irradiation was applied (Supplementary Fig. S6B).

Homing is the first step that is necessary for the successful engraftment of stem and progenitor cells into the bone marrow niche^21^. For the assessment of long-term homing and engraftment kinetics of AML1/ETO-expressing cells, B6 Albino mice were transplanted with Lin- cells obtained from constitutive Ubi-Luciferase 2 (Luc2) knock-in (Ubi-Luc2KI) mice and transduced with PINCO-GFP-AML1/ETO vector or the control vector (respectively Lin-LUC-AE and Lin-LUC-EV). Prior to transplantation, we determined that AML1/ETO-expressing Lin-LUC cells did not show any consistent difference in apoptotic rate as assessed by Annexin V/PI staining compared to vector-only trasduced cells (Supplementary Fig. S7). In order to determine the quantity and localization of the injected Luc2-positive cells, transplanted mice were analyzed by live bioimaging, initially daily and then weekly for 4 months. After 24 hours from injection, we observed a dramatic decrease in the luciferin signal of the transplanted cells in both experimental cohorts (Fig. 3C). Thereafter, luciferin signal increased in the control group, while the cohort of animals injected with Lin-LUC-AE cells showed a 24-hour delay in the recovery of luminescence (Fig. 3C and Supplementary Fig. S8 for extensive illustration), with a steady ten-fold difference in the signal intensity seen in spleens (*p*value = 0.00000661, Fig. 3D) and femurs (*p*value = 0.000005722, Fig. 3E) of recipient animals for the rest of the follow-up.

The transplantation of cells deriving from Ubi-Luc2KI mice into B6 Albino mice excludes the possibility of using CD45 allelic variants for tracking of transplanted cells. We devised a surrogate qPCR-based method to establish the percentage of donor cells in recipient tissues by quantifying the amount of *Luc2* gene normalized to nucleolin as described in Materials and Methods. Peripheral blood, bone marrow and spleen samples taken at the end of the engraftment experiment showed that the lower signal seen in animals engrafted with Lin-LUC-AE cells corresponded to a lower *Luc2* qPCR signal and hence to a lower number of cells and did not depend on a potential silencing of the Luc2 expression by AML1/ETO confirming the bioimaging results. The AML1/ETO cohort displayed a significantly lower engraftment than the control animals (Fig. 3F). Importantly, both lin-LUC-AE and Lin-LUC-EV cells gave rise to mature progeny as genomic DNA extracted from peripheral blood samples of all mice contained *Luc2* gene (Fig. 3F,G).

These experiments demonstrate that, regardless of the cell type and experimental approach used, AML1/ETO expression causes an impairment of homing and delays engraftment of HSPCs.

### EMT regulators are altered in cells expressing AML1/ETO

The increase in cell migration and decrease in adhesion observed in AML1/ETO expressing cells resembles the phenotype of Epithelial-To-Mesenchymal Transition (EMT), a process in which epithelial cells lose cell-to-cell attachment and acquire a motile phenotype that allows them to move to and colonize distant sites. EMT is physiologically relevant during organogenesis and is relevant to the progression of solid tumors (reviewed by Thiery *et al*.^22^), but little is known about its possible involvement in leukemogenesis.

**Figure 4:**
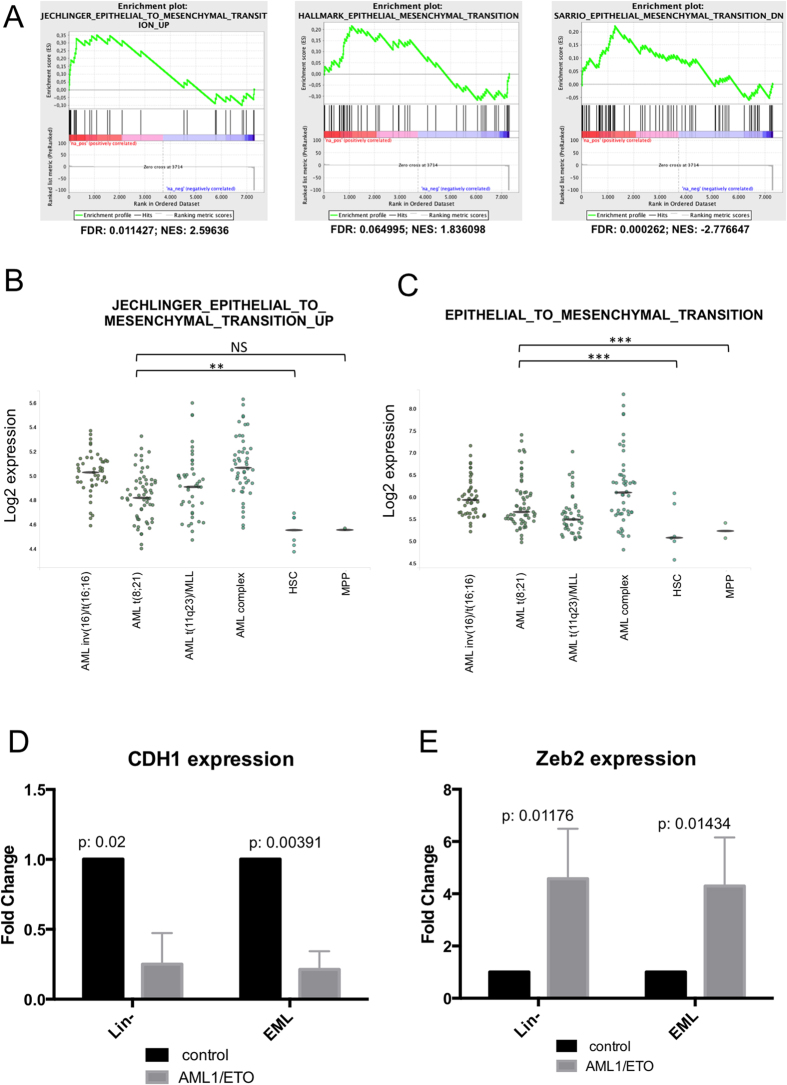
EMT regulators are altered in EML cells expressing AML1/ETO. (**A**) GSEA plot of EMT-related signatures in EML-EML-AE22 versus EML-EV control cells. FDR: False Discovery Rate; NES: Normalized Enrichment Score. (**B**,**C**) BloodSpot plots showing the expression data taken from different public EMT signatures in AML subtypes and healthy HSC/MPP cells. For each sample, the expression values in single datasets included in the analysis and the median value for each cohort are shown. (**D**,**E**) *Cdh1* (**D**) and *Zeb2* (**E**) expression levels were measured by RT-qPCR in Lin- with or without expression of AML1/ETO and in EML-EV and EML-AE22 cells. Levels are relative to *Tbp* expression and normalized for expression in the control cells.

We explored the possibility that AML1/ETO might induce an EMT-like process in AML blasts. GSEA performed on RNA-seq data obtained from EML-AE22 cells showed enrichment of at least three different EMT-related signatures (Fig. 4A), two of which were also overexpressed in t(8;21) AML according to meta-analysis using Bloodspot (Fig. 4B,C). Like the motility and adhesion functions, the EMT signatures are not specific to t(8;21) AML and are found also in CBF and complex karyotype AMLs.

In myeloid leukemias, expression of E-cadherin (CDH1) is downregulated due to an epigenetic shut-down of the gene^23^,^24^,^25^. Downregulation of epithelium-specific E-cadherin protein is considered the hallmark of EMT^26^. Therefore, E-cadherin expression was analyzed by RT-qPCR in EML clones expressing AML1/ETO, and murine Lin- cells after retroviral transduction with AML1/ETO (Lin-AE). Both models showed decreased *Cdh1* mRNA expression in AML1/ETO cells compared to their respective controls (Fig. 4D).

Next, EMT markers were analyzed in the EML cellular model, devoting particular attention to ZEB1, ZEB2, SNAI family (SNAI1, SNAI2, SNAI3) transcription factors and mesenchymal markers such as fibronectin and vimentin. Of these, *Zeb2* was significantly upregulated in Lin- and EML upon AML1/ETO expression (Fig. 4E). A possible activation of EMT-related pathways in AML was corroborated by the finding of an upregulation of several EMT-associated genes in AML-derived gene expression profiles. In particular, *ITGA5, SNAI1, S100A4, TCF3, ZEB2* were found to be upregulated in AML using the BloodSpot tool (Supplementary Fig. S9).

## Discussion

It is thought that leukemias derive from the transformation of a single HSPC and its progeny. The mechanisms that underlie the spreading of preleukemic and leukemic cells from the primary localization to the entire hematopoietic system are unknown. Not surprisingly, cell motility, cell-to-cell adhesion and tissue localization are functions that are consistently enriched in expression profiles obtained by microarray or RNA-sequencing of different AML model systems.

In this report we demonstrated that HSPC expressing AML1/ETO display an increase in cell migration *in vitro.* Coherently, silencing the fusion protein in Kasumi-1 and SKNO-1 patient-derived cell lines resulted in reduced motility. Adhesion to both primary bone marrow stroma and established stromal cell line was instead inhibited by AML1/ETO in all model systems. Importantly, *in vivo* experiments demonstrated that AML1/ETO-expressing cells display impairment in homing and slower engraftment kinetics following transplantation into recipient mice.

Two recent reports investigated the alterations of migration and adhesion caused by the expression of AML1/ETO. Ponnusamy *et al*.^27^ showed that in hematopoietic progenitor cells AML1/ETO epigenetically represses the *PSGL-1* gene that through an interaction with P-selectin plays a role in the rolling of hematopoietic cells on endothelial cells and their subsequent migration into tissues. PSLG-1 repression results in an impediment of cellular adhesion, which was restored after depletion of AML1/ETO. Furthermore, the AML1/ETO9a splice variant was shown to influence adhesion and migration of hemopoietic progenitor cells via the regulation of VLA-4 expression^12^. This confirms that the deregulation of adhesion molecules and of migratory properties is an important feature of t(8; 21) + AML.

The findings of the current study extend from these reports^12^,^28^ and describe the functional consequences of altered migration and adhesion in the form of impaired homing and delayed engraftment. The data suggest that HSPCs expressing AML1/ETO show abnormal interactions with the hematopoietic niche during the preleukemic phase, resulting in defective adhesion to stromal cells and increased motility. Alterations in the capacity to interact with the microenvironment are known to play a crucial role in tumor initiation and progression^28^.

The phenotype encountered bears similarities to EMT, a biological process that enable a polarized epiphelial cell to undergo complex biochemical changes that allow the cell to assume a mesenchymal phenotype, such as enhanced migratory ability, invasiveness, increased resistance to apoptosis and metabolic adaptation (reviewed in ref. 29). To date, a possible involvement of EMT in hematological tumors has not been sought directly, although altered expression of some of its modulators has been described^29^. A recent article showed that knockdown of ZEB1, an important EMT-related transcription factor, in MLL/AF9-driven leukaemia significantly reduced leukemic blast invasion^30^. Moreover, the authors identified several EMT-related genes significantly associated with poor overall survival of AML patients^30^. We propose that the machinery involved in EMT is deployed to achieve a more mobile and less adhesive phenotype in AML1/ETO-expressing cells. We believe that our study provides evidence in support of such a notion, albeit, the delineation of an exact mechanism requires further studies.

Our results suggest that abnormalities in the migration and adhesion properties of HSPCs expressing AML1/ETO are an early event, and may contribute to disease progression. The data shown here, do not exclude the possibility that the delayed engraftment is at least partially caused by a proliferative arrest/quiescence of AML1/ETO-expressing cells, as suggested by de Guzman *el al*.^31^. Of note, current evidence indicates that EMT induces stem cell-like properties in cells^32^. Stem cell properties include self-renewal and the capacity to remain quiescent likely linking our observations to the ones reported by de Guzman *et al*.^31^.

AML research has reached a high level of sophistication over the past decade or so but the refinement of standard therapies seems to lag behind^33^. Studies in solid tumors suggest that some of the known anti-cancer agents act through blocking EMT^34^,^35^ and further characterization of this process in AML may represent an important new therapeutic opportunity.

## Materials and Methods

### Primary cells

Lineage negative murine BM (Lin-) cells were purified from total bone marrow (BM) of 8 to 10-week old C57Bl/6 mice by negative selection using StemSep™ Mouse Hematopoietic Progenitor Cell Enrichment Kit (StemCell Technologies) according to the manufacturer’s instruction. After purification, lin- cells were infected with PINCO-GFP retrovirus and sorted for the expression of GFP as previously described^36^. For primary BM stroma, total BM from 8 to 10 week-old C57Bl/6 mice was plated in BM stromal medium (BMSM: IMDM, 12.5% FBS, 12.5% Horse Serum, 1 mM L-glutamine, 5 μM hydrocortisone, 50 μM β-mercaptoethanol) with for 3 weeks, changing the medium every 7 days.

### Cell lines and vectors

EML cell line (EML Cell Line, Clone 1 ATCC^®^ CRL-11691™), Kasumi-1, SKNO-1, AFT024 and 293T ecotropic cells were maintained in appropriate media as specified on the ATCC website.

EML-AE14 and EML-AE22 cell lines were generated by retroviral infection of EML cells with PINCO vector carrying full length AML1/ETO cDNA (PINCO-AE) or the control empty vector, and subsequent FACS sorting of GFP-positive cells. The EML PINCO-AE bulk population was subjected to serial dilution to select clones with high levels of AML1/ETO protein expression.

Kasumi-1 and SKNO-1 were infected with lentiviral pSICO vector expressing short interfering RNAs (shRNA) against the ETO moiety (cloned sequence: tgcaatgggatgtatgaattattcaggagatattcatacatcccattgcttttttc, where underlined portion is the target-specific sequence) and selected by puromycin treatment for 3 days (at a concentration respectively of 1 μg/mL and 7 μg/mL).

### Mice

Experiments involving mice were performed in agreement with the guidelines reported in^37^ and according to the international and Italian law. All procedures *in vivo* have been approved by the Italian Ministry of Health (protocol numbers: IEO 10/2013, TOP 1/2011) and performed under the supervision of the Institutional Committee for Animal Welfare. Mice used in the study were purchased from Charles River Laboratories: C57BL6/Ly5.2 (strain C57BL/6NCrl), C57BL6/Ly5.1 (strain *B6.SJL-PtprcaPepcb/BoyCrl*), B6 Albino mice (strain B6N-Tyrc-Brd/BrdCrCrl), NOD/SCID mice (strain NOD.CB17-Prkdcscid/NcrCrl). AML1/ETO-Rosa26-eYFP-Rosa26-cre-ER Ly5.2, Rosa26-Cre-ER Ly5.2 and Ubi-Luc2 knock-in mice were bread locally (IEO and University of Milan). We used 8- to 12-week-old males and females (average weight - 20–25 g).

### Myeloid differentiation

Myeloid differentiation experiments were performed as described by Tsai *et al*.^16^. Briefly EML cells and their derivatives were resuspended in IMDM supplemented with 20% horse serum, 12% WEHI 3B cell line conditioned medium, 8% BHK/MKL cell line conditioned medium, and 5 mM atRA (Sigma-Aldrich) for 3 days to produce a myeloid-committed population. Cells were then washed with buffered saline solution and cultured in IMDM supplemented with 20% horse serum and 10 to 50 ng/mL of murine GM-CSF (PeproTech) to induce myeloid differention at the myeloblast-promyelocite stage for 5 days. The differentiation status was assessed by FACS analysis of the surface markers.

### RNA-sequencing and computational analysis

Total RNA was extracted from 1 to 5 × 10^6^ cells using the RNeasy Mini Kit (Qiagen), quantified with Nanodrop (ThermoScientific) and reverse-transcribed using M-MuLV Reverse Transcriptase (Finnzymes). 10 ng of cDNA were used for amplification on the ABI 7500 machine. Primers used for RT-qPCR are listed in Supplementary Table S3. RNA-Seq library preparation from 1 μg of total RNA was performed with the TruSeq RNA Sample Prep kit (Illumina) according to the manufacturer’s instructions and sequenced on a Illumina HiSeq 2000 following standard protocols. Paired-end reads were aligned to the NCBI37/mm9 mouse reference genome using TopHat 1.3.1^38^. Differentially expressed genes were quantified using Cufflinks 2.2.1^38^, requiring minimum FPKM (fragments per kilobase of exon per million fragments mapped) of 2 in at least one experimental condition, p-value < = 0.05 and fold-change (FC) > = 1.5. Tracks for the UCSC genome browser^39^ were generated using the uniquely aligned reads. Tracks were linearly re-scaled to the same sequencing depth.

Gene Ontology (GO) enrichment analysis of differentially expressed genes was performed using both Ingenuity Pathway Analysis software (IPA) (IPA^®^, QIAGEN Redwood City; www.qiagen.com/ingenuity) and DAVID (https://david.ncifcrf.gov)^40^,^41^ with default parameters.

Gene set enrichment analysis (GSEA)^42^ was performed with pre-ranked algorithm and “classic” enrichment on all the genes having FPKM > = 2.

Raw dataset is available for download at the Gene Expression Omnibus (GEO) database (http://www.ncbi.nlm.nih.gov/gds) under the accession number GSE77185.

### Immunoblot

Cellular proteins were extracted in 1 × Laemmi Buffer (2% SDS, 10% glycerol, 5% 2-mercaptoethanol, 0.002% bromophenol blue and 60 mM Tris HCl, pH. 6.8), and 3 × 10^5^ lysed cells were loaded in each lane. Western Blot experiments were performed by standard methods using chemiluminescence detection system. The signal was acquired using Chemidoc XRS + Imager (Biorad) and analyzed with Image Lab software (Biorad). The following antibodies were used: anti-ETO C-20 (sc-9737, Santa Cruz), anti-vinculin (V9131, Sigma-Aldrich), anti-p15/16 C7 (sc-377412, Santa Cruz).

### Cell cytometry and FACS sorting

For the analysis of surface markers, anti-cKit-PE, anti-Sca-1-PE-Cy7, anti-Mac-1-PE-Cy7, anti-Gr1-PE-Cy7, anti-Ly5.2-APC (all from eBioscience) antibodies were used at a working dilution of 1:200 in PBS. Cells were stained according to standard methods. Signal was acquired by FACSCalibur or FACSCanto (BD Bioscience) and analyzed by Cell Quest software (BD Bioscience).

GFP-positive cells were sorted on MoFlo Astrios (Beckman Coulter) or FACSAria (BD Bioscience) flow sorters.

For apoptosis evaluation, cells were resuspended in annexin buffer (10 mM HEPES, 150 mM NaCl, 1mM MgCl_2_, 3.6 mM CaCl_2_, 5mM KCl) and stained with Annexin-APC (eBioscience) diluted 1:50 in annexin buffer for 1 hour. Cells were then washed and resupended in PI (eBioscience) 0.1 μg/mL in PBS buffer and acquired immediately at FACSCalibur or FACSCanto. Cell cycle was assessed using the BrdU incorporation (10 minute pulse) method following standard protocols.

### Migration assay

Migration assay was performed in Corning Transwell plates using 5 μm (primary Lin- cells or EML cell lines) or 8 μm (Kasumi-1 and SKNO-1) pore size. 2 × 10^5^ cells were plated in 200 μL of serum free medium in triplicate for each condition in the upper well, and 800 μL of complete medium were added to the lower chamber. Cell were left to migrate for 8 h at 37 °C, after which, cells from both chambers were collected separately and resuspended in 200 μL of PBS. Viable (trypan blue-negative) cells were counted using TC20^TM^ Automated Cell Counter (Biorad). Migration ratio was calculated as: “migrated cells/total cells plated”. For SDF1-dependent migration assay, 100 ng/mL recombinant murine SDF1β (250-20B, Peprotech) was added to the lower chamber at the beginning of the experiment.

### Adhesion assay

Twenty-four hours ahead of the assay, 25000 murine BM stromal cells or AFT024 cells were plated in 96-well plates to serve as cellular substrate. On the day of the assay, target cells were washed in PBS and stained with 0.5 μM CFSE (Thermo Fisher Scientific)/0.1% BSA in PBS for ten minutes at 37 °C on a rotating wheel. Following staining, cells were washed twice with BMSM or AFT024 medium to quench the CSFE, plated on the cellular substrate (100000 cells/well) and left to adhere for 2 h at 37 °C. After incubation, cells were washed three times with culture medium to remove unattached cells, lysed with 0.1% Triton-X and fluorescence was measured by Infinite^®^ 200 PRO reader (Tecan). Percentage of adherent cells was calculated as the ratio “Fluorescence of adhered cells”/”Fluorescence of total cells plated” after background signal correction.

### Homing assay

For non competitive homing assay, 30 × 10^6^ EML-EV or EML-AE22 cells were stained with eFluor^®^670 Cell Proliferation Dye (eBioscience) according to manufacturer instructions, resuspended in 500 μL of PBS and injected *i.v.* into NOD-SCID mice irradiated (3 Gy) 12 h before injection. Three mice were injected per cell type. After 16 h, mice were sacrificed, and eFluor^®^670-positive cells were enumerated in the BM and spleen of each animal by cell cytometry.

Competitive homing assay was carried out using AML1/ETO-Rosa26-eYFP-Rosa26-cre-ER Ly5.2 knock-in mice along with Rosa26-Cre-ER Ly5.2 mice as control. Both strains were fed with tamoxifen-containing food for 3 weeks to induce AML1/ETO recombination and thus gene expression in all of the hematopoietic system. Mice were sacrificed, mononucleated BM cells were isolated using Histopaque 1083 (Sigma Aldrich), and subjected to FACS sorting for live/eYFP-positive (AML1/ETO-Rosa26-eYFP-Rosa26-cre-ER cells) or live (Rosa26-cre-ER) cells. Sorted cells were mixed in equal parts, and the percentage of the two cell types in the resulting mix was determined by cell cytometry. 4 × 10^6^ total cells were injected into the caudal vein of irradiated (6.5 Gy) or non-irradiated recipient C57BL6/Ly5.1 mice. 3 mice were used for each group. After 16 h, recipient mice were sacrificed, target organs were processed and the obtained cells were stained for Ly5.2 and analyzed by cell cytometry. AML1/ETO knock-in and control cells were distinguished of the basis of eYFP expression.

### Bioimaging assay

Hematopoietic progenitor cells (Lin-) expressing the firefly luciferase (Luc2, Promega, Wisconsin) were obtained from the Ubi-Luc2KI transgenic mouse (TOP srl, Italy). This model was generated by knock-in insertion in a proprietary locus of a transgene constitutively expressing Luc2 in all tissues including the hemopoietic system. The biosensor was flanked by matrix attachment region (MAR) sequences insulating the reporter gene from the influence of the surrounding chromatin thus nullifying position effects (European patent No. EP 1298988B1; US patent No. 7943815). Lin- cells were transduced with PINCO-GFP-AE or control vector and sorted for GFP positivity. 5 × 10^5^ cells were injected into the caudal vein of lethally irradiated (6.5 Gy) B6 Albino mice. Animals were injected intraperitoneally with 80 mg/kg luciferin (Promega) 15 minutes prior the imaging session. Mice were anaesthetized using Isofluorane (Isofluorane-Vet, Merial) and kept under anesthesia during the 5 minutes of the imaging session carried out with a CCD-camera performing both ventral and dorsal acquisitions (Xenogen IVIS Lumina System: Caliper, PerkinElmer). The dose of luciferin and the time selected for its complete biodistribution were experimentally established^43^,^44^; the parameters were set in order to measure bioluminescence in the linear range of the emission curve. Photon emission in selected body areas was measured using the Living Image Software (Caliper, PerkinElmer). Bioluminescence was measured in specific regions of interest (ROIs) manually selected with the aid of a grid. Data were expressed as photon/second/cm^2^/steradiant (p/s/cm^2^/sr). The intensity of bioluminescence was color-coded for visualization purposes.

### Engraftment analysis

A surrogate SybrGreen (Applied Biosystems) qPCR-based method was used for the assessment of engraftment in tissues of transplanted animals at the end of the bioimaging assay taking advantage of the fact that whilst nucleolin (*Ncl*) gene is present in all cells, *luc2* is only present in donor cells genomic DNA. An artificial series of samples containing growing percentage of Ubi-Luc2KI lin- cells added to B6 Albino lin- cells was prepared and genomic DNA was extracted. *Luc2* and *Ncl* genes were amplified and *Luc2* gene PCR signal was normalized to *Ncl* PCR signal. Primer sequence is given in Supplementary Table S3. In order to obtain a calibration curve, normalized PCR signal and the corresponding percentage of Ubi-Luc2KI cells were plotted and the linear fit was found. This resulted in a formula that allowed for the estimation of Luc2-bearing cells in experimental samples (Supplementary Fig. S10).

### Statistical analysis

Statistical analysis was performed by GraphPad Prism 6 software (San Diego, CA, United States). Data are the results of three independent experiments. All results were expressed as mean ± SE. Statistical significance was calculated with two-sided t-test.

## Additional Information

**How to cite this article**: Saia, M. *et al*. AML1/ETO accelerates cell migration and impairs cell-to-cell adhesion and homing of hematopoietic stem/progenitor cells. *Sci. Rep.* **6**, 34957; doi: 10.1038/srep34957 (2016).

## Supplementary Material

Supplementary Information

Supplementary Information
