# Supplementary material for: AML1/ETO accelerates cell migration and impairs cell-to-cell adhesion and homing of hematopoietic stem/progenitor cells
Source: Sci Rep. 2016 Oct 7;6:34957. doi: 10.1038/srep34957 (PMC5054523; doi:10.1038/srep34957)

Supplementary figures

AML1/ETO accelerates cell migration and impairs cell-to-cell adhesion and homing of hematopoietic stem/progenitor cells

Marco Saia, Alberto Termanini, Nicoletta Rizzi, Massimiliano Mazza, Elisa Barbieri, Debora Valli, Paolo Ciana, Alicja M. Gruszka, Myriam Alcalay

Supplementary Figure 1. Surface marker profile of EML cells analysed by flow cytometry according to stemness and lineage markers. Bars represent the percentage of positive cells for each surface marker analyzed.

Supplementary Figure 2. Characterization of cell cycle, apoptosis and senescence of AML1/ETO-expressing EML clones compared to vector control cells. (A) Cell cycle analysis by BrdU incorporation. (B) Annexin V / propidium iodide apoptosis assay. (c) The determination of no senescence onset upon expression of AML1/ETO as determined by Western blot analysis of p16 expression. p53KO murine embryonic fibroblasts (MEF) were used as a positive control.

Supplementary Figure 3. Comparative analysis of quantitative PCR (qPCR) versus RNA-seq of 16 genes detected in EML-AE22 cells versus control EML cells. Dashed lines correspond to -1 and +1 fold change.

Supplementary Figure 4. (A). Examples of motility and adhesion-related genes in EML-AE22 RNA-seq dataset compared to EML-EV control cells classified according to their function. (B) Enriched pathways analyzed by DAVID in EML-AE22 RNA-seq dataset compared to EML-EV control cells. Red squares indicate the motility, adhesion and cytoskeleton related pathways. (C) Ingenuity Pathway Analysis (IPA) of enriched functions in U937-AE microarray data compared to U937 control cells.

Supplementary Figure 5. (A) Transwell migration assay of EML-AE14 and EML-EV control cells with 100 ng/mL SDF1 or without (PBS control). (B) Adhesion assay of EML-AE14 and EML-EV control cells performed on murine primary stroma or on AFTY024 stromal cell line.

Supplementary Figure 6. (A) Competitive homing assay of AML1/ETO-eYFP-Cre-ER Ly5.2 (AE-YFP) and Cre-ER Ly5.2 control cells transplanted into lethally irradiated (6.5 Gy) recipient mice. Percentages of the two populations mixed before transplantation is also shown (pre-transplantation mix). Bone marrow and spleen bars represent the relative percentage of each cell type out of the total transplanted Ly5.2 cells enumerated by flow cytometry. (B) Percentage of total Ly5.2-positive cells detected in the organs of non-irradiated or irradiated Ly5.1 recipient mice upon homing.

AML1/ETO accelerates cell migration and impairs cell-to-cell adhesion and homing of hematopoietic stem/progenitor cells

Marco Saia, Alberto Termanini, Nicoletta Rizzi, Massimiliano Mazza, Elisa Barbieri, Debora Valli, Paolo Ciana, Alicja M. Gruszka, Myriam Alcalay

Supplementary Figure 7. Annexin V-Propidium Iodide analysis to asses apoptosis in Lin-Luc2 cells transduced with AML1/ETO (Lin-LUC2-AE) or vector only (Lin-LUC2-EV). Live cells: Annexin $V^{-}PI^{-}$, Early apoptosis: Annexin $V^{+}PI^{-}$, Late apoptosis: Annexin $V^{+}PI^{+}$, Cell Debris: Annexin $V^{-}PI^{+}$

Supplementary Figure 8. Homing and engraftment of Lin-LUC-EV (control) and Lin-LUC-AE (AML1/ETO transduced) cells into B6 Albino mice. Five mice were used for each cohort. Both dorsal and ventral acquisitions are shown. Mouse number 10 died 72 hours after injection. Images were acquired every 24 hours from transplantation.

Supplementary Figure 9. Bloodspot plots showing the expression levels of five EMT-related genes (*ITGA5, SNAI1, S100A4, TCF3, ZEB2*) in public microarray data derived from human AML subtypes and normal HSC/MPP cells.

Supplementary Figure 10. Calibration curve for the estimation of Ubi-Luc2KI lin- cells mixed with B6 Albino lin- cells.

Supplementary Figure S1

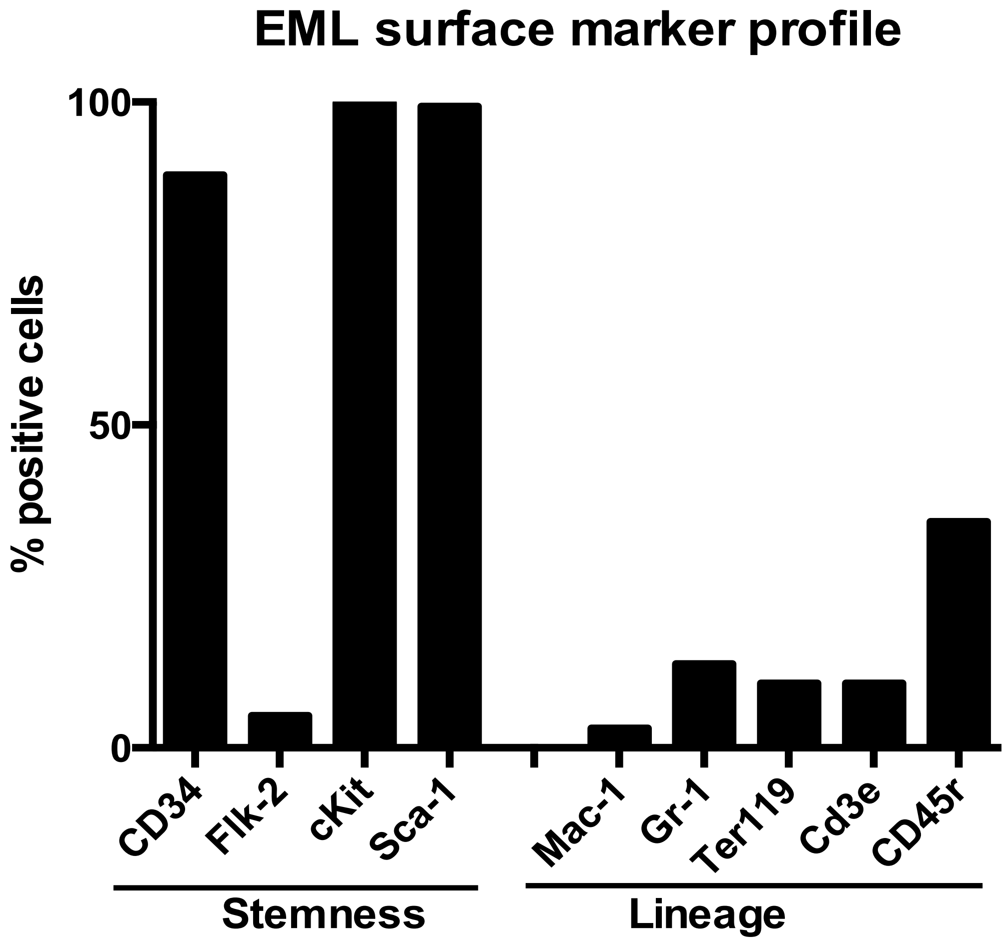

Supplementary Figure S2

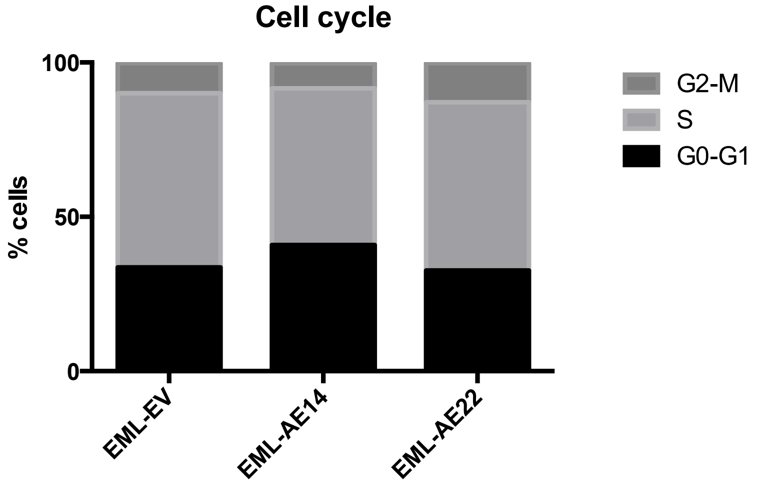

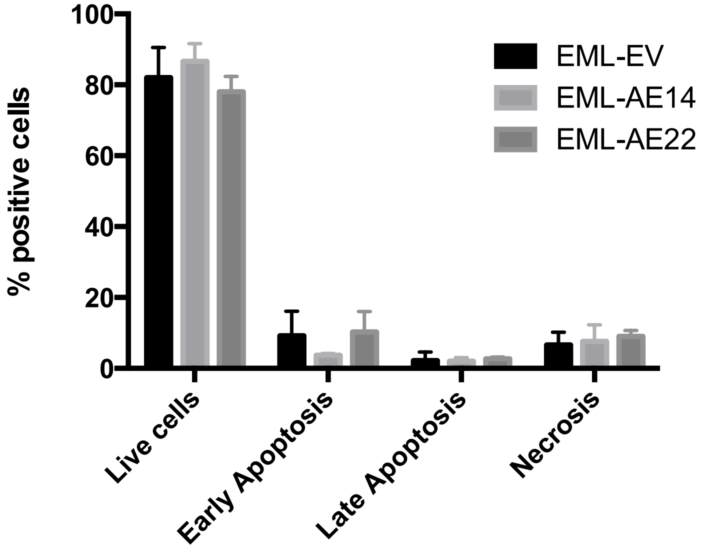

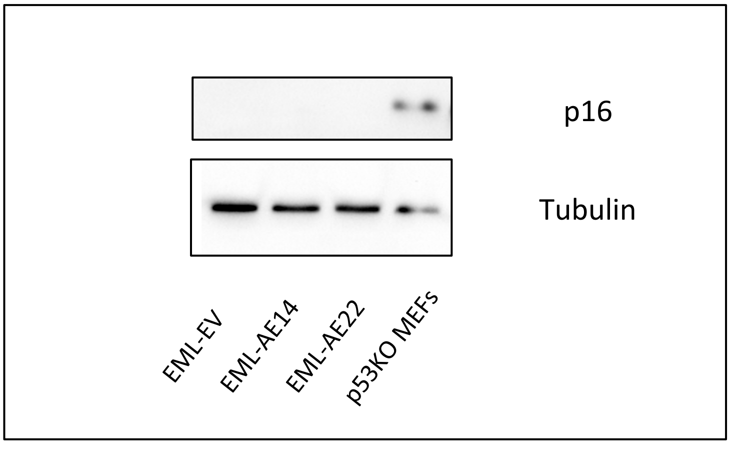

Supplementary Figure S3

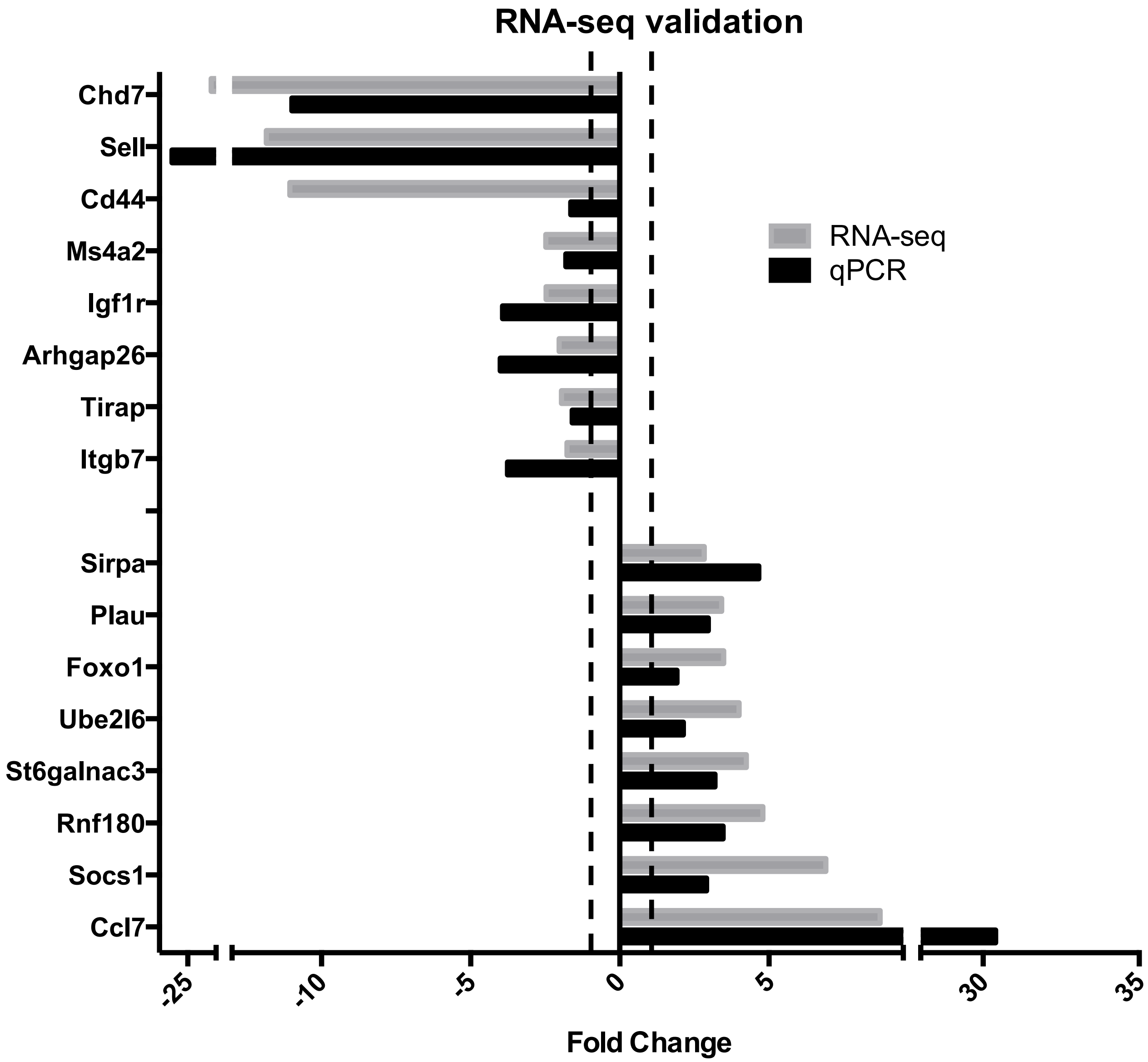

Supplementary Figure S4

A

Functional classes	Regulated Genes
**Chemokines and receptors**	
	CCL2, CCL7, CCR1, CCR5, CXCR4
**Adhesion molecules**	
	CD34, CD44, CD93, ITGA2, ITGA4, ITGA5, ITGB7, SELL, ICAM1
**Motility related signalling proteins**	
	RHOB, SMAD3, VAV3, PAK1, PRKAA1, TRIO, SRGAP2, PIP5K1C
**Cytoskeleton related proteins**	
	GSN, JAKMIP1, SSH3, NCKAP1L, DIAP3, ACTR6, TRIOBP, AFAP1, CAPG, MYLIP

B

Term	RT	Genes	Count	%	P-Value
Lysosome	RT		28	1,9	1,0E-7
p53 signaling pathway	RT		17	1,2	3,2E-5
Graft-versus-host disease	RT		15	1,0	6,3E-5
Type I diabetes mellitus	RT		15	1,0	1,6E-4
Allograft rejection	RT		14	1,0	2,5E-4
Antigen processing and presentation	RT		18	1,2	3,2E-4
Natural killer cell mediated cytotoxicity	RT		21	1,4	6,0E-4
Hematopoietic cell lineage	RT		16	1,1	1,2E-3
Fc gamma R-mediated phagocytosis	RT		17	1,2	2,1E-3
Biosynthesis of unsaturated fatty acids	RT		8	0,5	2,9E-3
Viral myocarditis	RT		16	1,1	3,7E-3
Gap junction	RT		15	1,0	4,1E-3
NOD-like receptor signaling pathway	RT		12	0,8	5,4E-3
Autoimmune thyroid disease	RT		13	0,9	6,3E-3
Fc epsilon RI signaling pathway	RT		14	1,0	7,0E-3
Apoptosis	RT		14	1,0	1,1E-2
MAPK signaling pathway	RT		31	2,1	1,3E-2
Cytokine-cytokine receptor interaction	RT		29	2,0	1,3E-2
Endocytosis	RT		25	1,7	1,4E-2
Chemokine signaling pathway	RT		23	1,6	1,5E-2
Regulation of actin cytoskeleton	RT		26	1,8	1,8E-2
Phosphatidylinositol signaling system	RT		12	0,8	2,2E-2
Jak-STAT signaling pathway	RT		19	1,3	3,2E-2
Cell adhesion molecules (CAMs)	RT		19	1,3	3,6E-2
Pathways in cancer	RT		33	2,3	5,6E-2

C

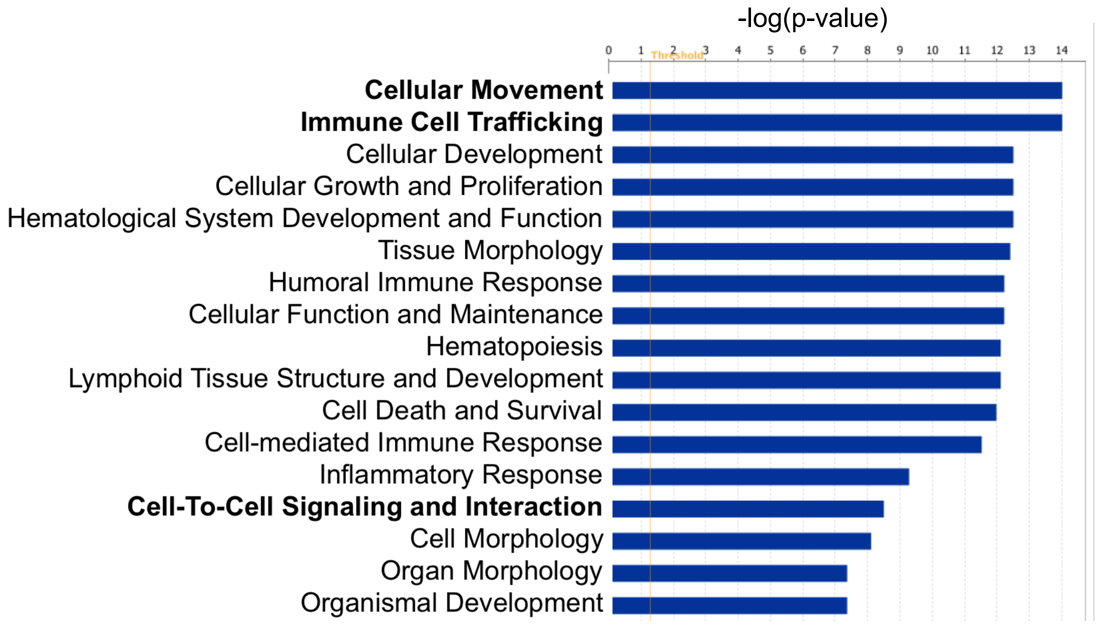

Supplementary Figure S5

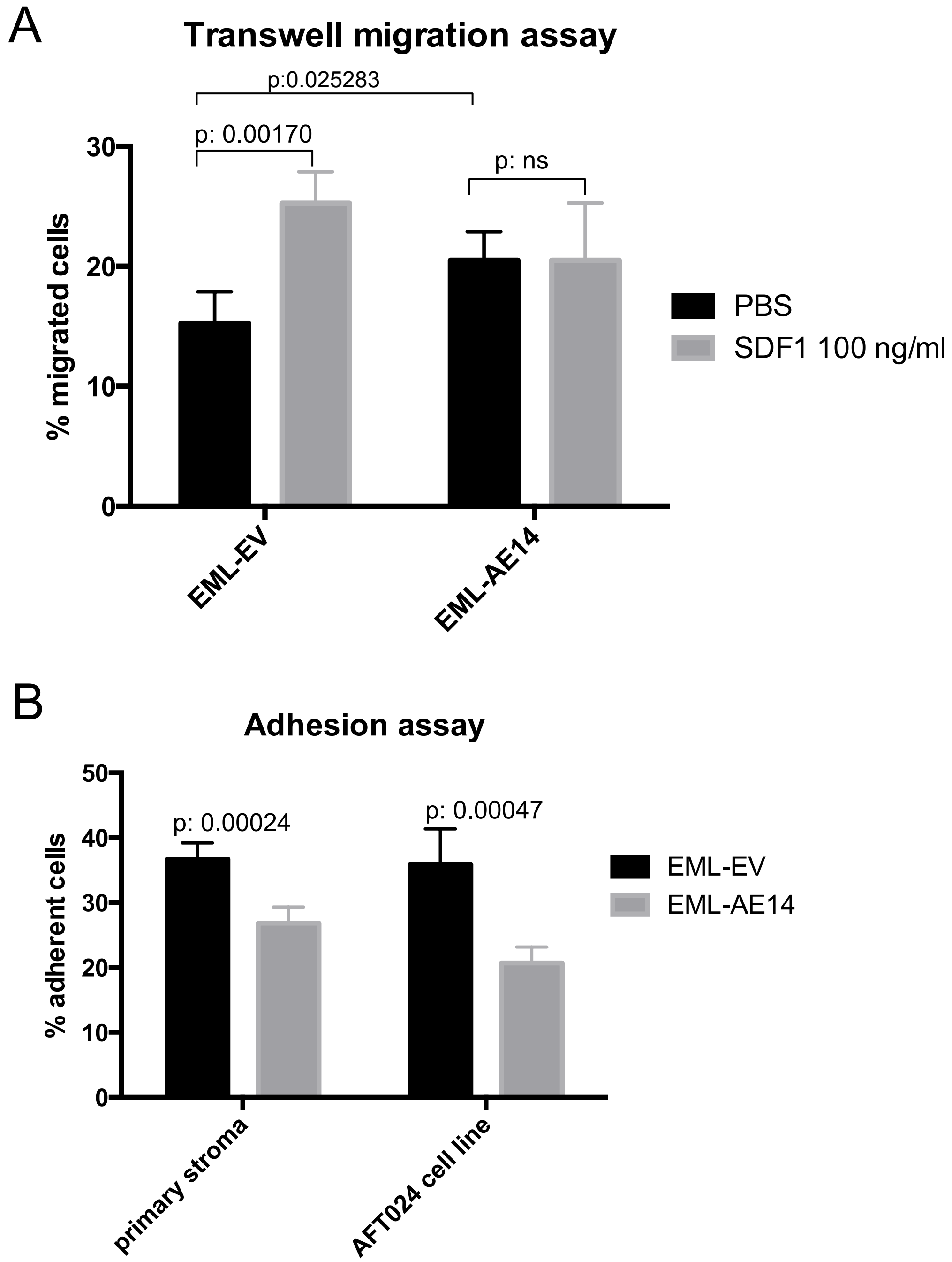

Supplementary Figure S6

A

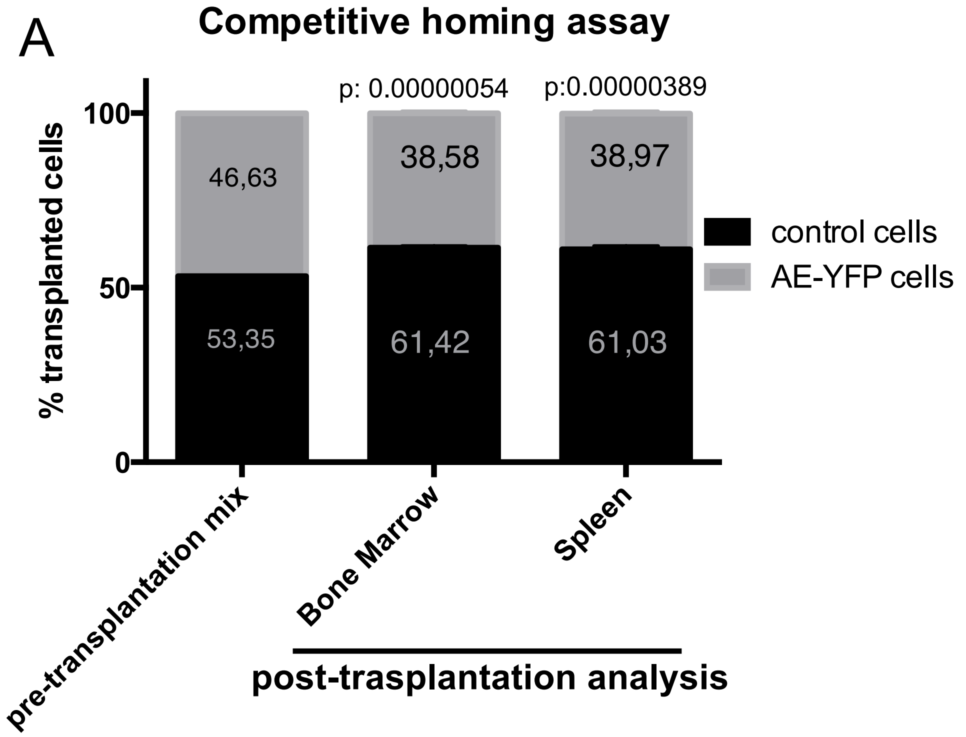

B

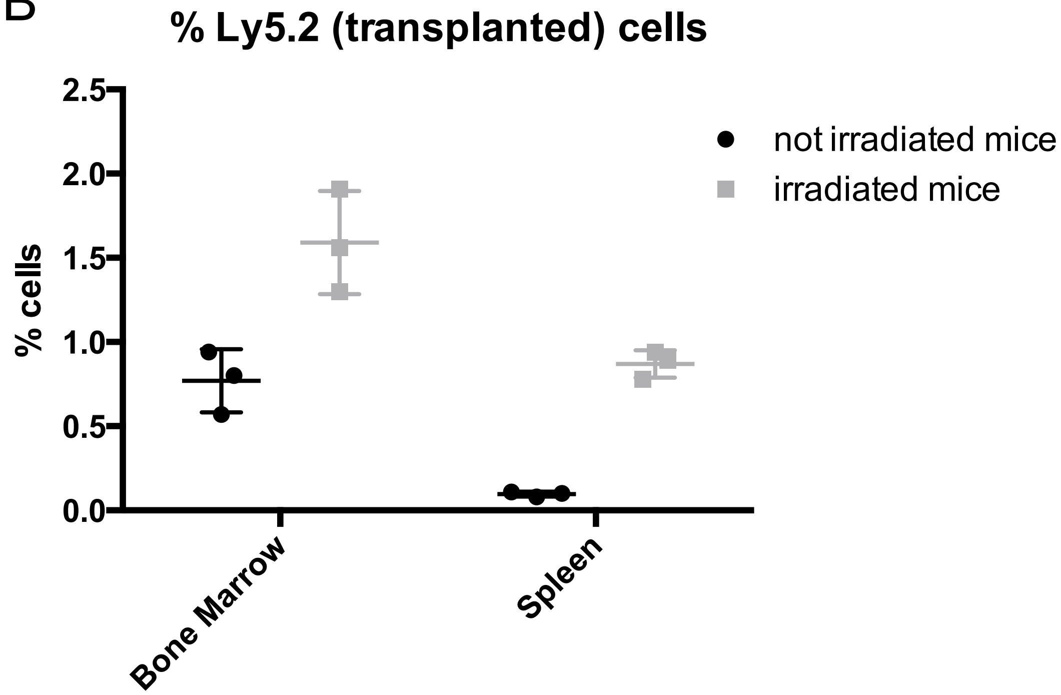

Supplementary Figure S7

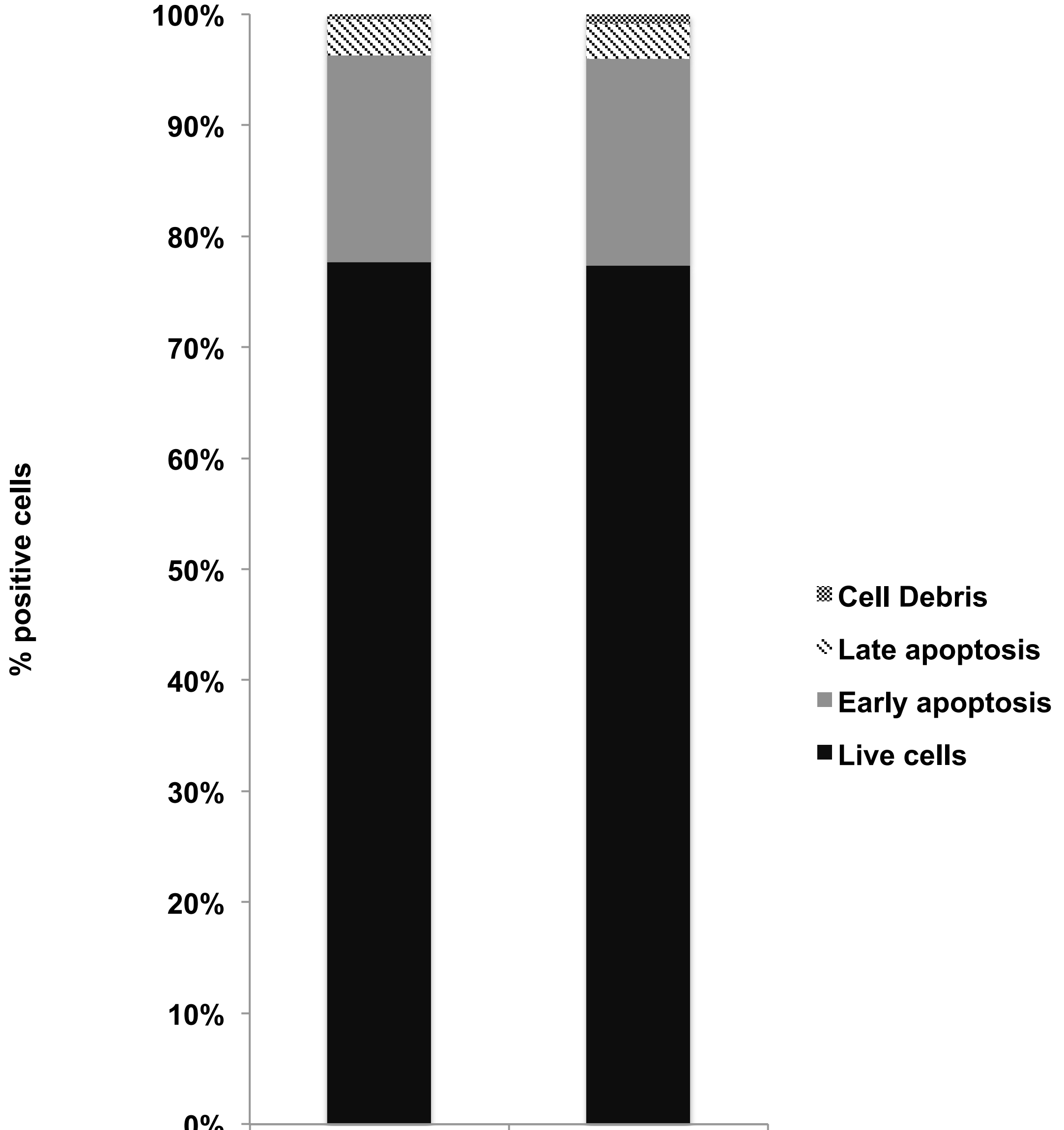

	Lin-Luc2-EV	Lin-Luc2-AE
Cell Debris	0.5	0.9
Late apoptosis	3.2	3.1
Early apoptosis	18.6	18.6
Live cells	77.6	77.2

Supplementary Figure S8

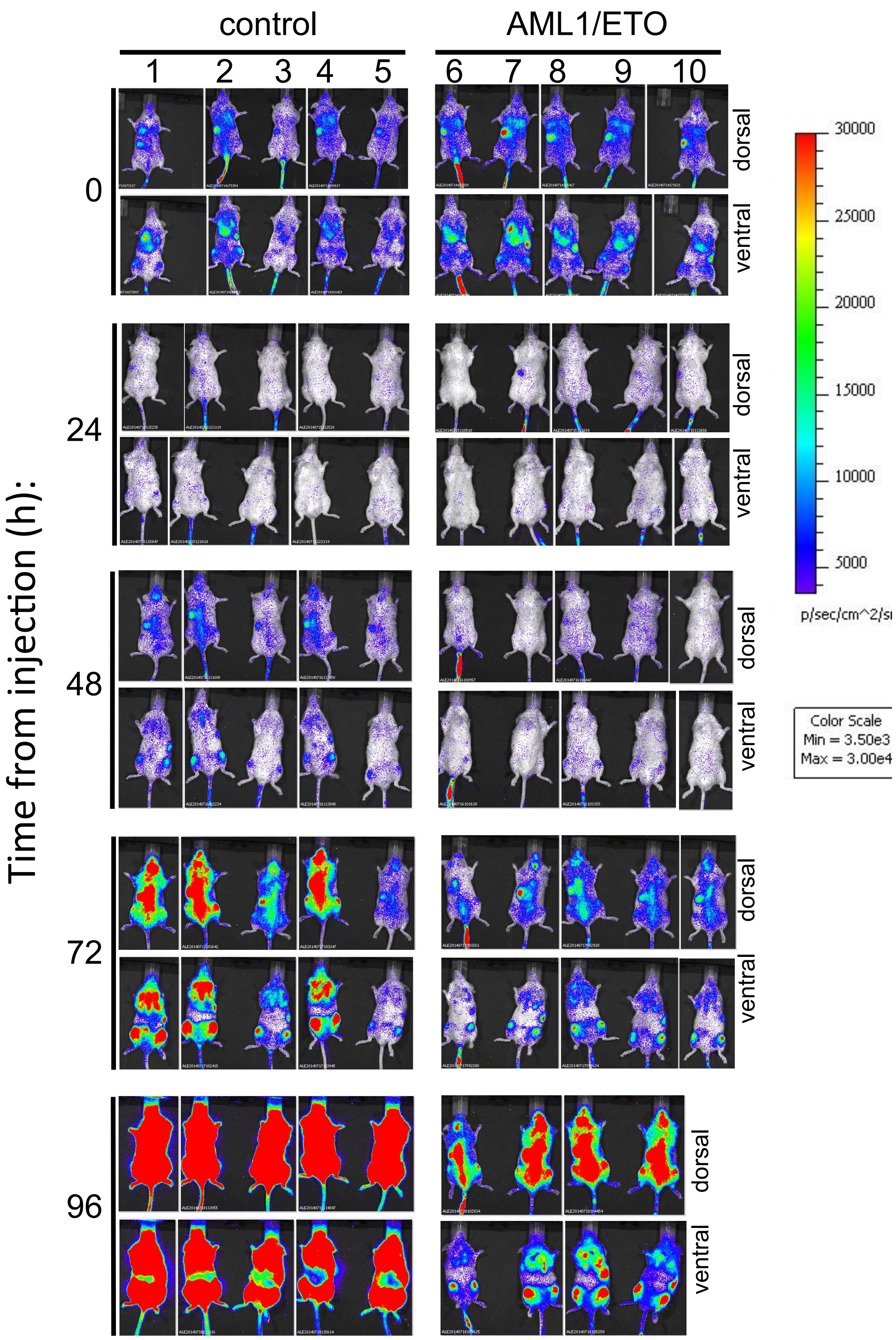

Supplementary Figure S9

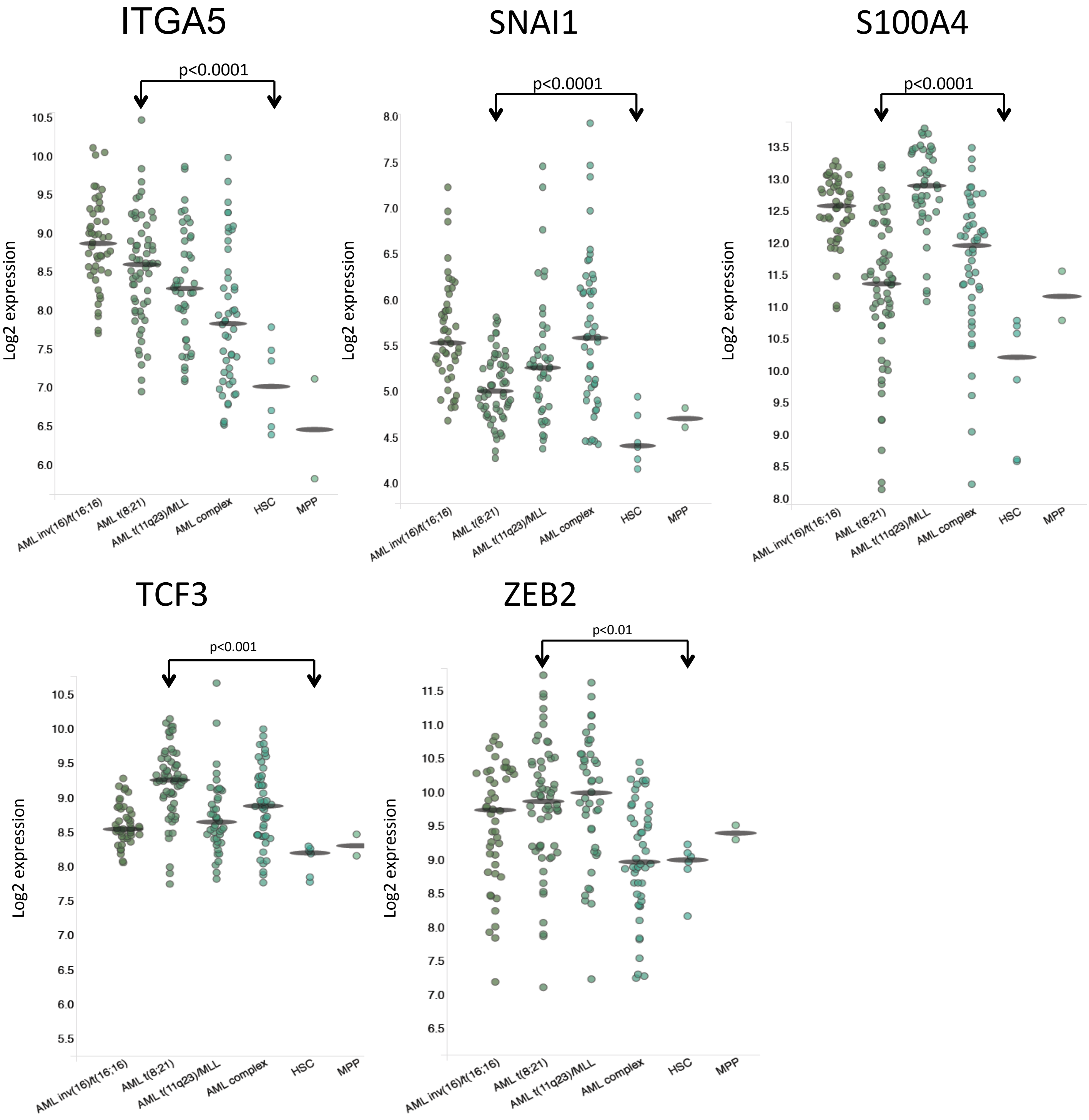

Supplementary Figure S10

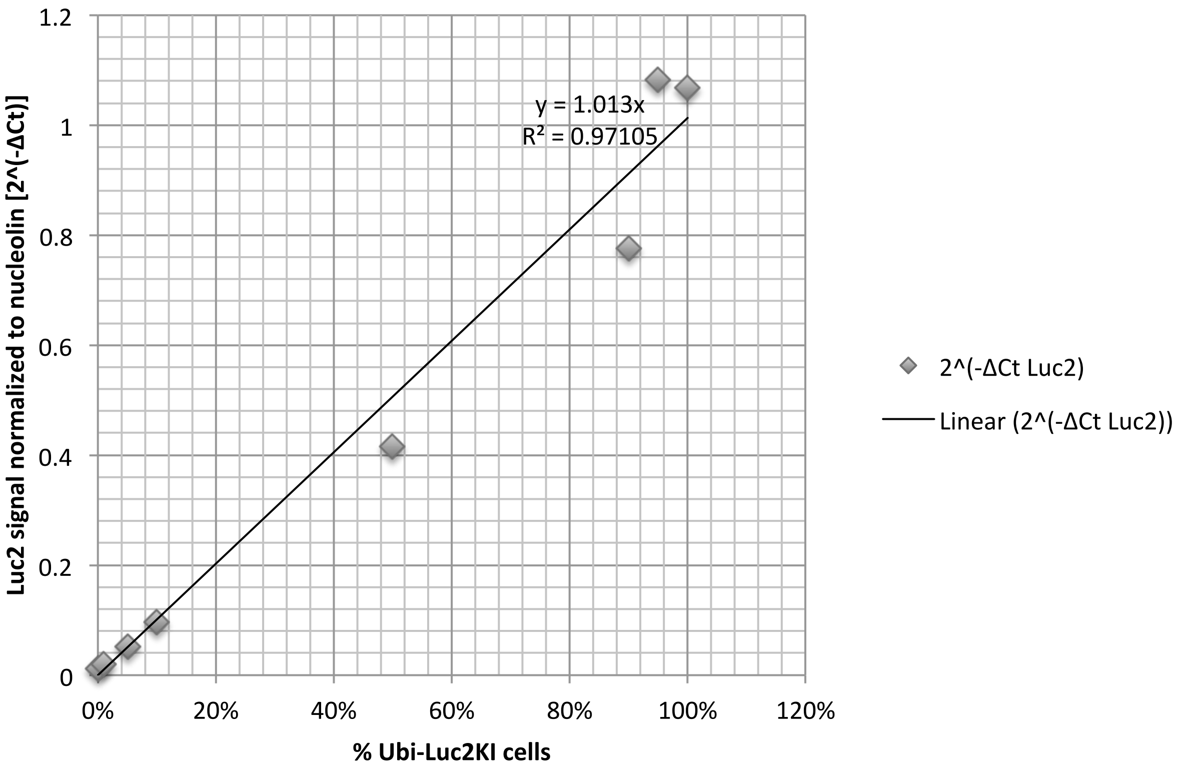

Supplement: Supplementary Information [file srep34957-s1.pdf]
